# A Review of the Role of Flavonoids in Peptic Ulcer (2010–2020)

**DOI:** 10.3390/molecules25225431

**Published:** 2020-11-20

**Authors:** Catarina Serafim, Maria Elaine Araruna, Edvaldo Alves Júnior, Margareth Diniz, Clélia Hiruma-Lima, Leônia Batista

**Affiliations:** 1 Postgraduate Program in Natural Products and Bioactive Synthetic, Health Sciences Center, Federal University of Paraiba, João Pessoa 58051900, Paraiba, Brazil; catarinaalvesdelima@gmail.com (C.S.); elaine.araruna@gmail.com (M.E.A.); edvaldojunioralves@gmail.com (E.A.J.); 2 Department of Pharmacy, Health Sciences Center, Federal University of Paraíba, João Pessoa 58051900, Paraiba, Brazil; margareth@reitoria.ufpb.br; 3 Department of Structural and Functional Biology (Physiology), Institute of Biosciences, São Paulo State University, Botucatu 18618970, São Paulo, Brazil; clelia.hiruma@unesp.br

**Keywords:** flavonoids, peptic ulcer, review

## Abstract

Peptic ulcers are characterized by erosions on the mucosa of the gastrointestinal tract that may reach the muscle layer. Their etiology is multifactorial and occurs when the balance between offensive and protective factors of the mucosa is disturbed. Peptic ulcers represent a global health problem, affecting millions of people worldwide and showing high rates of recurrence. *Helicobacter pylori* infection and the use of non-steroidal anti-inflammatory drugs (NSAIDs) are one of the most important predisposing factors for the development of peptic ulcers. Therefore, new approaches to complementary treatments are needed to prevent the development of ulcers and their recurrence. Natural products such as medicinal plants and their isolated compounds have been widely used in experimental models of peptic ulcers. Flavonoids are among the molecules of greatest interest in biological assays due to their anti-inflammatory and antioxidant properties. The present study is a literature review of flavonoids that have been reported to show peptic ulcer activity in experimental models. Studies published from January 2010 to January 2020 were selected from reference databases. This review refers to a collection of flavonoids with antiulcer activity in vivo and in vitro models.

## 1. Introduction

Peptic ulcer is an acid-peptic disease characterized by the rupture of the protective barrier of the epithelial mucosa of the esophagus, stomach or duodenum [1,2]. The etiology of this condition is considered complex and multifactorial, comprising an imbalance between the protective and aggressive agents of the gastric mucosa [3]. The main mucosal defense factors include the synthesis of cytoprotective prostaglandins (PGs), mucus, bicarbonate (HCO_3_^−^), nitric oxide (NO), endogenous antioxidant system, and adequate blood flow [4,5]. Aggressive agents include hydrochloric acid (HCl) secretion, and pepsin activity, bile reflux [6], abnormal motility [7], decreased blood flow and infection with *Helicobacter pylori*, a bacterium that frequently colonizes the human stomach [8,9,10].

**Figure 1:**
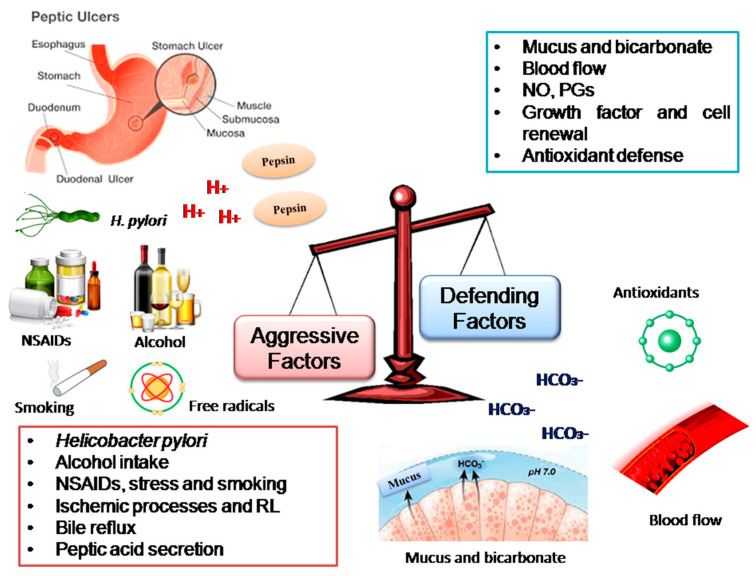
Schematic representation of peptic ulcer etiopathogenesis.

In addition to endogenous aggressive factors, the development of ulceration has been associated with some exogenous factors such as inappropriate eating habits [11,12], stress [13] and chemical agents such as alcohol [14], smoking [15] and prolonged and excessive use of non-steroidal anti-inflammatory drugs (NSAIDs) [16] (Figure 1).

Peptic ulcer is a common disease that develops in 5 to 10% of the world population at least once during the individual’s lifetime, with an incidence of 0.1–0.3% per year [2]. The infection by *H. pylori* bacteria and the use of NSAIDs are considered to be of the most important predisposing factors for the development of peptic ulcers [17,18]. Studies indicate that about 90% of duodenal ulcers and 70% of gastric ulcers are associated with *H. pylori* [19] and NSAIDs may be responsible for 50% of ulcer formation [20].

Peptic ulcer therapy aims to relieve pain, heal the ulceration, and prevent complications and recurrence. The current treatment of peptic ulcers consists mainly of anti-secretory medications, such as histamine H_2_ receptor antagonists and proton pump inhibitors (PPIs) and cytoprotectors (sucralfate and salts bismuth); in cases where *H. pylori* infection occurs, the use of antimicrobials is necessary [21]. The development of PPIs represented a therapeutic advance for peptic ulcers. However, the pharmacological treatment of peptic ulcers still has some limiting factors, such as side effects, especially in individuals on prolonged therapy and the increased resistance of *H. pylori* to antimicrobial agents [22]. Besides, they present poor healing of ulcers and recurrence of ulcerations, generating a high economic impact for users and public health systems, mainly due to complications such as bleeding and perforation [23].

From this perspective, the gastroprotective and antiulcerogenic potential of many medicinal plants and their isolated constituents has been investigated to develop complementary treatments to alleviate the severity of ulcerative diseases and prevent recurrence episodes [24]. Medicinal plants have historically proven their value as a source of molecules with therapeutic potential. In addition, many of its constituents have served as inspiration and starting points for medicinal chemistry and the development of new molecules and drugs [25]. Thus, in recent decades, studies on plant metabolites have increased [26].

Flavonoids consist of a class of secondary metabolites from medicinal plants. They are a very diverse group of polyphenolic compounds widely distributed in nature and are recognized as the pigments responsible for the colors of the leaves. These compounds’ phytochemicals also play important roles in resistance to pathogens and predators, protection of UV radiation, and heat [27]. All flavonoids have a basic 15-carbon skeleton (C_6_-C_3_-C_6_), based on the flavylium nucleus, which consists of three phenolic rings (A, B, and C) [28].

Flavonoids can be classified into different groups according to the chemical characteristics of the molecules. Among these, flavonols, flavones, flavanones, isoflavones, flavanols (catechins) and anthocyanidins are the best known. In addition, flavan-3-ois or flavan-3,4-diols give rise to the various structural types of the condensed tannin class [28]. The various classes of flavonoids differ in the level of oxidation and pattern of substitution of ring C, while individual compounds within the same class differ in the pattern of substitution of rings A and B [29].

The structural diversity of flavonoids is derived by substitutions in their base skeleton through hydroxylation, glycosylation, methylation, acylation, and prenylation reactions in different positions. These constituents are present in plants as aglycone or glycoside conjugates, but most flavonoids are present in the form of glycosides under natural conditions. The attached sugar portions include d-glucose, l-rhamnose, rhamnose-glucose, galactose, lignin, and arabinose [30].

Flavonoids present many pharmacological activities including cardioprotective [31], neuroprotective [32] and anti-inflammatory [33]. In addition, it has been reported that these constituents act in the gastrointestinal tract [34], exerting antispasmodic [35], anti-diarrheal [36], anti-secretory and anti-ulcer effects [37] and intestinal anti-inflammatory [38].

This class of compounds has become increasingly popular in terms of health protection because it has a remarkable spectrum of biochemical and pharmacological activities. Perhaps the most active area of research with flavonoids today is the possible medicinal contribution that flavonoids make to human health [39].

In a previous article, this research group reviewed the action of flavonoids with anti-ulcer activity until 2009 [37]. Thus, this work came with the proposal to renew and contribute to the knowledge in this area, to provide relevant information on the main findings of the last ten years and explore what has changed from the last review until today. Based on this, the present study aimed to review the literature on the role of flavonoids in gastroprotection in different experimental models of peptic ulcer between the years 2010 and 2020.

## 2. Results

The flavonoids selected for this study were based on their effects in experimental models of peptic ulcer against induction with harmful agents. This article provides information on the chemical structure, natural and/or food source, experimental models (in vivo or in vitro), dose, route of administration, test organism, effect, and the mechanisms of action.

### 2.1. Flavonols

Flavonols are chemically characterized by the presence of a carbonyl group in position 4, a hydroxyl group in position 3, and a double bond between positions 2 and 3. The main representatives of this group are kaempferol and quercetin [29,40,41].

#### 2.1.1. Kaempferol

**Figure 2:**
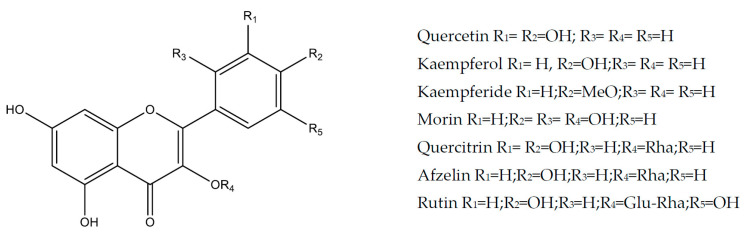
Flavonols with gastroprotective and/or anti-ulcer activity.

Kaempferol (3,5,7-trihydroxy-2-(4-hydroxy-phenyl)chromen-4-one) (Figure 2) is a flavonol that is commonly found in many foods (broccoli, cabbage, beans, leeks, tomatoes, strawberries and grapes) and vegetable products commonly used in traditional medicine such as *Ginkgo biloba*, *Moringa oleifera* and propolis [42]. Several studies have demonstrated the wide range of pharmacological activities of this flavonoid, such as antioxidants [43], cardioprotective [44] and anticancer [45].

Kaempferol (40, 80 and 160 mg/kg, p.o.) and the control drug omeprazole (20 mg/kg) protects gastric ulcers induced by ethanol in mice, inhibiting the accumulation of neutrophils, decreasing the activity of myeloperoxidase (MPO) and the levels of pro-inflammatory cytokines such as TNF-α, IL-1β and interleukin-6 (IL-6), improving NO and gastric mucus [46]. Kaempferol also inhibits the growth of *H. pylori* in vitro with a 0.05 mMol/L of minimum inhibitory concentration (MIC); this effect involves the expression of the cytotoxin-associated gene A (CagA) and vacuolating cytotoxin A (Vac A) genes and reduces the inflammatory process triggered by this microorganism (reduced levels of the cytokines TNF-α, IL-1β and interleukin-8 (IL-8) in AGS cells) [47].

Omeprazole is a proton pump inhibitor, the final target of the acid secretion cascade, that allows for prolonged inhibition of gastric acid secretion [22].

#### 2.1.2. Kaempferide

Kaempferide (3,5,7-trihydroxy-2-(4-methoxy-phenyl)chromen-4-one) (Figure 2) is an O-methylated flavonol derivative of kaempferol. This compound isolated from green propolis prevented the ulcer induced by HCl/ethanol intraperitoneally and indomethacin by oral route (3 mg/kg), using carbenoxolone as a positive control (200 mg/kg, p.o.), as well as the ulcer induced by pylorus ligature (positive control, omeprazole 20 mg/kg, p.o.) in mice. Gastroprotection was accompanied by normalization of superoxide dismutase (SOD), catalase (CAT) and glutathione transferase (GST) activities and reduced MPO activity. In addition, it increased the gastric mucin content and decreased the volume, pH, total acidity and pepsin activity of the gastric juice [48].

Carbenoxolone is a natural product derived from glycyrrhizic acid extracted from the roots of *Glycyrrhiza glabra* that has gastric cytoprotective activity, acting on the maintenance of the mucus layer [49].

#### 2.1.3. Quercetin

Quercetin (2-(3,4-dihydroxyphenyl)-3,5,7-trihydroxychromen-4-one) (Figure 2) is a flavonol found mainly in fruits and vegetables such as onions, broccoli, apple, cherry and grape and has anti-inflammatory, antioxidant, anti-hypertensive, vasodilatory, anti-hypercholesterolemic, anti-atherosclerotic, anti-tumor and antiviral effects [50]. Over the years, many researchers have studied the gastroprotective potential of quercetin in vivo [51,52]. The gastroprotective properties of nano-quercetin (2.5 mg) in vivo were evaluated in the ethanol-induced ulcer model in rats. The results obtained suggest a blockage of the synthesis and secretion of matrix metalloproteinase 9 (MMP)-9, as well as, of the infiltration of inflammatory cells and oxidative damage. Besides, the quercetin regulates apoptosis and the activity of cyclooxygenase (COX) and nitric oxide synthase (NOS) [53]. Quercetin (50 mg/kg, p.o., rats) and the standard drug famotidine (50 mg/kg), inhibitor of H₂-type histamine receptors, showed a gastroprotective effect on indomethacin-induced acute gastric ulcers; this effect is related to its antioxidant potential (increased activity of antioxidant enzymes) [54]. Quercetin protects the gastric mucosa of rats (50 or 100 mg/kg, p.o) and Caco-2 cells (0.0331 mMol/L) against oxidative stress and inflammation induced by indomethacin, involving increased nuclear translocation of the nuclear factor related to erythroid 2 (Nrf_2_), as well as the activities of superoxide dismutase (SOD) and glutathione peroxidase (GPx). Quercetin also prevented indomethacin-induced nuclear factor kappa B (NF-κB) activation and ICAM-1 and P-selectin expression [55]. With its gastroprotective potential already proven in previous studies, the effect on *H. pylori* in vitro (0.212–0.423 mMol/L) and in vivo (25 mg/kg) of quercetin was investigated, showing promising results, involving the reduction in the levels of inflammatory cytokines tumor necrosis factor-α (TNF-α) and interleukin-1β (IL-1β) when compared with untreated mice [56].

To enhance the activity of some substances that already have a proven gastroprotective effect, some studies have evaluated the use of combinations of conventional drugs with these substances. Examples of these combinations are quercetin with famotidine and pantoprazole [57,58].

#### 2.1.4. Morin

Morin (2-(2,4-dihydroxy-phenyl)-3,5,7-trihydroxy-chromen-4-one) (Figure 2), a member of the flavonols, is characterized by a yellowish pigment isolated from Chinese herbs in the *Moraceae* family, and exhibits hepatoprotective, cardioprotective and neuroprotective effects through antioxidant and anti-inflammatory mechanisms [59]. Morin (50 mg/kg, p.o., rat) protected gastric mucosal against indomethacin-induced damage by downregulating MPO, NF-κB, TNF-α, inducible nitric oxide synthase (iNOS), ICAM-1, IL-6 and caspase-3, as well as, upregulating prostaglandin E_2_ (PGE_2_) and SOD [60].

#### 2.1.5. Rutin

Rutin, also called quercetin-3-rutinoside (Figure 2), is a flavonoid found in tomatoes, orange, carrots, sweet potatoes, black tea, and apple peels. The name rutin comes from the plant *Ruta graveolens*, which also contains rutin. Chemically, it is a glycoside that comprises flavonolic quercetin aglycone together with rutinosis disaccharide. It has some pharmacological activities described in the literature, such as neuroprotective, cardioprotective, and anti-inflammatory [61]. The mechanism underlying these effects is believed to stem from its antioxidant activity and the ability to suppress processes mediated by free radicals, inferring that the antioxidant properties of rutin may be useful in the clinical treatment of gastric disorders [62]. Some studies have also evaluated its effects on the gastrointestinal tract. Rutin (200 mg/kg, p.o.) showed gastroprotective activity against gastric lesions induced with indomethacin in rats, inhibition of neutrophil infiltration, and suppression of the generation of oxidative stress (increasing GSH and SOD and reducing MPO) [63]. Rutin (50, 100, and 200 mg/kg, p.o., rats) was evaluated in the ischemia and reperfusion model showing inhibition of iNOS activity, MPO activity and levels of malondialdehyde (MDA) in the gastric mucosa [64]. The anti-ulcerogenic potential of rutin (20, 40, and 80 mg/kg, p.o.) has already been investigated in several ulcer induction models: ethanol, stress and acetic acid in rats reduced gastric damage in all models, with decreased MDA levels and increased GPx activity [65]. Another investigation provides information on the molecular mechanism of the action of rutin with inhibition of proton pump activity (IC_50_ of 0.0590 mMol/L) [66].

#### 2.1.6. Quercitrin and Afzelin

Quercitrin is a glycoside formed from the flavonoid quercetin and afzelin (Figure 2) is a glycosyloxiflavone that is kaempferol attached to an osidic unit at position 3 via a glycosidic bond, extracted from the plant *Solidago chilensis* (Asteraceae), popularly known as “Brazilian arnica”, and widely used in folk medicine to treat gastric disorders [67]. The gastroprotective effects of these two flavonoids were evaluated in the models of acute gastric ulcer induced by HCl/ethanol in mice, using carbenoxolone (200 mg/kg) as a positive control. Quercitrin (1.38 mg/kg, p.o.) and afzelin (0.026 and 0.078 mg/kg, p.o.) showed gastroprotective activity in this model. Quercitrin prevented the depletion of gastric glutathione (GSH) content and quercitrin and afzelin reduced MPO activity. These compounds also inhibited H^+^-K^+^-ATPase activity at a concentration of 0.00223 to 0.222 mMol/L [68].

### 2.2. Flavanols

Flavanols are characterized by the hydroxyl group in position 3. The main members of this class are catechin and epicatechin [29].

#### Catechin, Epicatechin, Epigallocatechin Gallate and Derivates

**Figure 3:**
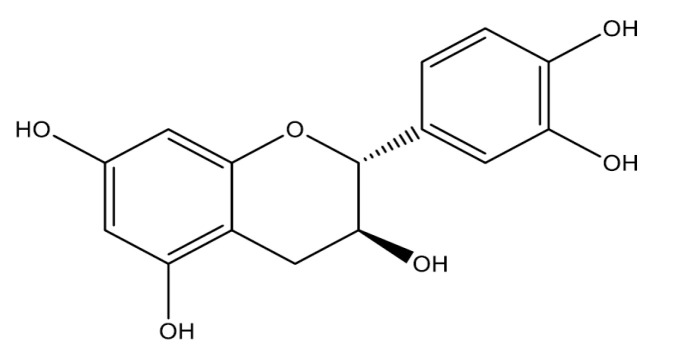
Flavanols (catechin) with gastroprotective and/or anti-ulcer activity.

Catechin (Figure 3) and its gallate derivatives are monomeric flavanols with potent antioxidant and anti-inflammatory activities present in green tea [69]. Some studies have demonstrated the gastroprotective effect of catechin and their derivates. Epigallocatechin gallate (0.5, 1, 2, 3, and 5 mg/kg, p.o., rat) has anti-ulcer activity in the NSAIDs model, related to its ability to reduce important inflammatory mediators and oxidative stress present in pathological conditions of ulceration. The mechanism associated with this effect involves the reduction in NO and iNOS levels, neutrophil infiltration, and MPO activity. In addition, to modulating the imbalance of pro-inflammatory and anti-inflammatory cytokines. Mucin content in gastric tissue and levels of PGEs have also been reported to protect the mucosa [70,71]. Epicatechin (25 and 50 mg/kg, p.o., rat) has a gastroprotective action in models of ethanol (carbenoxolone, 100 mg/kg, positive group) and NSAIDs (cimetidine, 100 mg/kg, positive group), involving the participation of sulfhydryl compounds (SHs), via adrenergic, K_ATP_ channels, increased mucus production and decreased H^+^ secretion. Immunohistochemistry demonstrated the involvement of SOD, NO, and heat shock protein-70 (HSP-70) in gastroprotection [72]. Catechin (35 mg/kg, rat, p.o.) increased the activity of intracellular antioxidant enzymes glutathione peroxidase (GPx), glutathione reductase (GR) and total sulfhydryl and up-regulating of a Nrf2 in the NSAIDs model in vivo and in vitro [73]. The anti-secretory potential of catechin derivatives plays a crucial role in the protective capacity of green tea against peptic ulcer, which is mediated by the inhibition of H^+^-K^+^-ATPase activity. The inhibitory function of *H. pylori* infection is another pharmacological mechanism of the catechin derivates and other constituents of green tea for the treatment of peptic ulcers [74,75,76].

Cimetidine, a histamine H_2_ receptor antagonist, is commonly used in the treatment of gastric lesions and as a pharmacological tool in experimental studies [77].

### 2.3. Flavones

Flavones have a carbonyl group in position 4 and a double bond between positions 2 and 3. However, unlike flavonols, they do not have hydroxyl in position 3 [29].

#### 2.3.1. Baicalein

**Figure 4:**
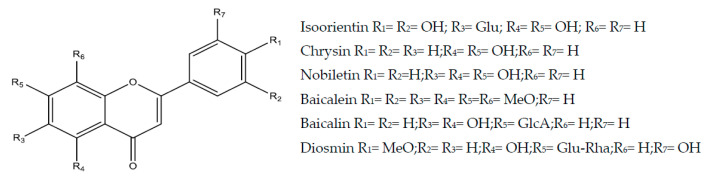
Flavones with gastroprotective and/or anti-ulcer activity.

Baicalein (5,6,7-trihydroxy-2-phenylchromen-4-one or 5,6,7-trihydroxyfavone) (Figure 4) is a flavone found mainly in the root of *Scutellaria baicalensis* with antioxidant [78], anti-cancer [79] and antimicrobial potential [80]. This flavone exhibited a gastroprotective effect against lesions induced by acidified ethanol and pylorus ligature in mice (10, 30, and 100 mg/kg, p.o.). The mechanism related to this effect involves the participation of SHs, adrenergic pathway, COX, NO, and K_ATP_ channels. This effect was evidenced since the administration of blockers of these pathways (*N*-ethylmaleimide, yohimbine, indomethacin, *N*-w-nitro-l-arginine methyl ester hydrochloride, and glibenclamide, respectively) caused a reversal of the gastroprotective effect of baicalein. Besides, baicalein showed cytoprotective effects by increasing the secretion of gastric mucus, antioxidants with increased levels of GSH, and reduced activity of MPO and antisecretory with the inhibition of H^+^-K^+^-ATPase activity (0.30370 and 0.111 mMol/L) in vitro [81]. The gastroprotective effects of baicalein involve inhibition of *Helicobacter pylori* in vitro (IC_50_ 0.331 mMol/L) and in vivo. The molecular events involved with this effect involve the suppression of the expression of the Vac A gene, the decrease in the capacity of adhesion and invasion of this microorganism and the reduction in the levels of the IL-8 cytokine in AGS cells. In the in vivo model, with infection of mice, treatment with baicalein inhibited the growth of *H. pylori* in the stomach of these animals and the serum levels of IL-1β and IgM and IgA specific for *H. pylori* were reduced [82].

#### 2.3.2. Baicalin

Baicalin is a flavone glycoside; it is the glucuronide of baicalein (Figure 4). The gastroprotective and antisecretory effects of baicalin (5, 10 and 15 mg/kg, p.o., rats) were evaluated by different induction methods such as alcohol, stress, NSAIDs and pyloric ligation. These involved Nfr2 modulation [83] and inhibition of *H. pylori* in vitro (IC_50_ 0.431 mMol/L) and in vivo [82,84], involving the reduction in the expression of the Vac A gene, the ability of adhesion and invasion of the bacteria and the levels of IL-8 and IL-1β. Another effect of baicalin against *H. pylori* is the inhibition of the urease enzyme (IC_50_ 0.82 mMol/L) in gastric epithelial cells (GES-1). This finding was confirmed by the study of molecular docking. In addition, baicalin also exerted an inhibitory effect of *H. pylori* on multi-drug resistant strains, significantly reducing the MICs of the amoxicillin and tetracycline antimicrobials. This effect was related to reduced expression of the hefA gene [84].

In addition, the Zinc–Baicalin (BA–Zn) complex was evaluated in the acetic acid model in rats, showing significant anti-inflammatory and antioxidant effects. BA-Zn (6.5 and 13 mg/kg) increased SOD activity and GPx level and reduced the MDA content and IL-8 and TNF-α levels in the gastric mucosa [85].

#### 2.3.3. Chrysin

Chrysin (5,7-dihydroxy-2-phenylchromen-4-one or 5,7-dihydroxyfavone) (Figure 4) is a naturally occurring flavone present in several plants (*Passiflora caerulea*, *Passiflora incarnate*, *Oroxylum indicum*, *Matricaria chamomilla*) and natural products such as propolis [86]. Its molecule is very similar to that of other flavonoids such as apigenin and luteolin [87]. Anti-cancer, anti-inflammatory, antioxidant, and anti-hypercholesterolemic effects have been reported in the literature [88]. Chrysin (50 and 100 mg/kg, p.o.) showed a gastroprotective effect in the indomethacin-induced gastric ulcer model in rats, involving cytoprotective mechanisms (promoting mucus secretion), anti-inflammatory (inhibition of pro-inflammatory cytokines and macrophage mobilization), antioxidant (participation of GSH, CAT, and MDA) and angiogenic with increased expression of vascular endothelial growth factor (VEGF) and basic fibroblast growth factor (FGF). Omeprazole (30 mg/kg) was used as a control group [89]. In addition, chrysin (10, 50 and 100 mg/kg, p.o.) and carbenoxolone (100 mg/kg, positive control) protected the gastric mucosa from ethanol-induced injury in mice [90]. Recently, Fagundes et al., (2020) [90] demonstrated the gastric healing effect of chrysin (10 mg/kg, p.o., mice) and lansoprazole (30 mg/kg, control group) in the acetic acid model, improving the expression of inflammatory genes such as COX-2, inhibiting the negative remodeling promoted by MMP-9, increasing the cell proliferation effect via epidermal growth factor (EGF) and reducing cell apoptosis by modulating caspase-3.

#### 2.3.4. Diosmin

Diosmin (synonym 3,5,7-trihydroxy-4-methoxyflavone 7-rutinoside or diosmetin 7-rutinoside) (Figure 4) is a natural flavonoid glycoside obtained from different plant species constituted by a flavone named diosmetin, a unit of glucose and a unit of rhamnose. Diosmin exhibited antioxidant and anti-inflammatory properties, attenuating liver, kidney, and heart damage in experimental models [91]. Diosmin showed gastroprotection (100 mg/kg, p.o.) against ethanol-induced gastric ulcers in rats, suppressed gastric inflammation, reducing TNF-α and NF-kB expression and restored levels of anti-inflammatory interleukin-10 (IL-10). It interrupted gastric oxidative stress through the inhibition of lipid peroxides with a reduction in the activity of MPO and a concomitant increase in GSH, GPx, and total antioxidant capacity (TAC). These actions were associated with positive regulation of gastric cytoprotective PGE_2_ and NO. Regarding apoptosis of the gastric mucosa, it suppressed the activity of caspase-3 and cytochrome C (Cit_C_) with B-cell lymphoma-2 (Bcl-2) enhancement [92]. Diosmin derivates inhibit *H. pylori* in vitro and in silico models (CIM 0.822 mMol/L) [93]. Diosmin nanoparticles (20 mg/kg) PLGA-optimized (polyd-lactide-co-glycolide) and coated with chitosan were also evaluated for anti-ulcer activity in the rat ethanol model, showing satisfactory results [94].

#### 2.3.5. Isoorientin

Isoorientin (synonym luteolin-6-C-glucoside) (Figure 4), a type of flavone C-glycoside, extracted from plant species such as *Gentiana triflora* and *Eremurus spectabilis*, exhibits several biological properties such as analgesic [95], neuroprotective [96] and hepatoprotective [97]. The gastroprotective activity of isoorientin was evaluated in the model of gastric damage induced by indomethacin in rats (25, 50 and 100 mg/kg, p.o.) showed significant anti-ulcer effect with a reduction in the level of MDA and increased SOD activity and GSH levels. Famotidine was used as a standard anti-ulcer medication [98].

#### 2.3.6. Nobiletin

Nobiletin (2-(3,4-dimethoxyphenyl)-5,6,7,8-tetramethoxychromen-4-one) (Figure 4) is a polymethoxylated flavonoid found in citrus fruits, with anti-inflammatory [99], hepatoprotective [100], neuroprotective [101] and anticancer [102] properties. Nobiletin (5, 10, or 20 mg/kg, p.o.) and cimetidine (100 mg/kg, positive group) has protective effects on gastric ulcers induced by absolute ethanol in mice by stimulating cytoprotective PGE_2_, antioxidant enzymes (SOD) and negatively regulating the expression of pro-inflammatory cytokines (TNF-α and IL-6) [103].

### 2.4. Flavanones

Flavanones are characterized by the presence of the carbonyl group in position 4, for example, naringin and hesperidin [29].

#### 2.4.1. Naringin

**Figure 5:**
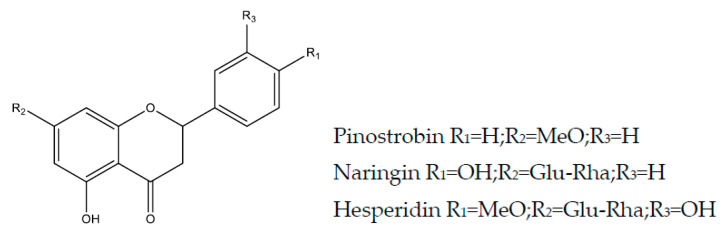
Flavanones with gastroprotective and/or anti-ulcer activity.

Naringin (synonym 4′,5,7-trihydroxyflavanone7-ramnoglucoside or naringenin 7-neoesperidoside) (Figure 5) is a flavanone that occurs naturally in citrus fruits, responsible for the bitter taste of the fruit, and which has a wide range of pharmacological activities including anti-inflammatory, anticancer, effects on bone regeneration, metabolic syndrome, oxidative stress and central nervous system [104]. Previous studies had already reported the anti-ulcer activity of naringin [105,106]. Recently, naringin (100 and 200 mg/kg, p.o.) was evaluated against ethanol-induced ulcers in rats and showed a significant reduction in mucosal damage, gastric MDA level, gastric expression of TNF-α, caspase-3 and IL-6 with elevated gastric GSH and SOD [107].

#### 2.4.2. Pinostrobin

Pinostrobin (2S-5-hidroxy-7-metoxy-2-phenyl-2,3-di-hydrocromen-4-one) (Figure 5) is flavanone obtained from *Boesenbergia rotunda* (*Zingiberaceae*) used for the treatment of gastrointestinal disorders, including peptic ulcer. These constituents present diverse biological activities, including antioxidant, anti-inflammatory, antiviral, and anticancer properties [108]. In the ethanol-induced ulcer model in rats, it protected the gastric mucosa, with inhibitory effects in COX-2 enzyme (20 and 40 mg/kg, p.o.), with omeprazole (20 mg/kg) as a positive control [109].

#### 2.4.3. Hesperidin and Neohesperidine

Hesperidin and neohesperidine (Figure 5) are flavanones found in citrus products, such as orange and lemon, associated with anti-inflammatory, antimicrobial, and anticancer effects [110]. Several researchers have examined the gastroprotective activity of these flavanones in experimental ulcer models [111,112,113]. Chinese decoction containing hesperidin reduced gastric ulceration indomethacin and ethanol induced [114,115]. Hesperidin and neohesperidine (100 mg/kg, p.o., rat) were evaluated in the NSAIDs model. Ulcer index, expression of gastric COX-2 gene, TNF-α, MDA, and GSH content in the stomach were measured [116]. Hesperidin (150, 300, and 450 mg/kg, p.o., rat) and omeprazole (20 mg/kg) were also evaluated in NSAIDs and stress models, with GSH, SOD, and CAT investigation [117]. The synergistic effect of a combination of lycopene and hesperidin (100 mg/kg, p.o., rat) has been reported on ulcers induced by pyloric ligation [118]. Hesperidin (50 mg/kg, p.o., rat) and omeprazole (20 mg/kg) exhibit protective effects in ethanol-induced peptic ulcer. Expression of gastric COX-2 gene, TNF-α, MDA, the content of hydrogen peroxide groups (H_2_O_2_), and Thiol (-SH) in the stomach and antioxidant enzymes SOD, CAT and GPx were measured. Hesperidin decreased COX-2 expression and gastric DNA fragmentation, reduced TNF-α production, and gastric lipid peroxidation [119]. Hesperidin (100 mg/kg, p.o.) prevents oxidative cell damage in the stress model in rats by significantly increasing levels of GSH, SOD, CAT, and mucin levels in the gastric mucosa and decreasing levels of lipid peroxidation and inflammatory markers [120]. Hesperidin (3 and 10 mg/kg, p.o., rat) has a protective effect on acetic acid-induced chronic gastric ulcers (assessment of mucin, GSH, SOD, CAT, and MPO levels) [121]. Hesperidin also inhibited *H. pylori* in vitro (0.05–0.5 mMol/L) [122].

### 2.5. Isoflavonoids

Isoflavonoids have ring B attached to the rest of the molecule through carbon 3, instead of being attached to carbon 2. An example of this class is genistein [29].

#### 2.5.1. Genistein

**Figure 6:**
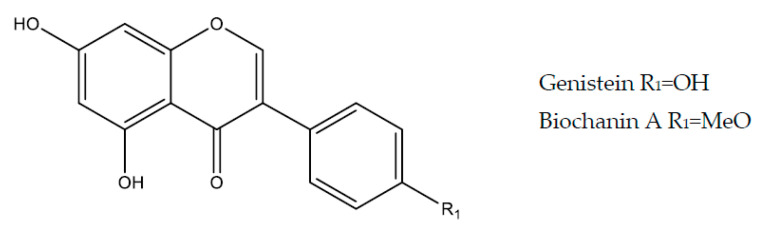
Isoflavonoids with gastroprotective and/or anti-ulcer activity.

Genistein (5,7-dihydroxy-3-(4-hydroxyphenyl)chromen-4-one) (Figure 6) belongs to a natural isoflavonoids class that has an estrogenic effect, being one typical example of a phytoestrogen compound [123]. It was isolated for the first time from *Genista tinctoria* and was named after the genus of this plant, but today it is known that the main sources of genistein are soy-based foods (*Glycine max* or *Soy hispida*) [124]. Genistein (10 mg/kg, p.o., rat) showed a gastroprotective effect in an experimental model of gastric lesion induced by indomethacin, reducing inflammation (TNF-α), decreasing oxidative stress (increased SOD and reducing MDA and iNOS activity) and restoring mucoprotective function (PGE_2_ levels) [125]. Another study evaluated the protective effect of genistein (10 mg/kg, p.o., rat) in the experimental model of gastric lesion induced by indomethacin in a 7-day treatment protocol. The animals were treated with genistein once a day for seven days before the induction of gastric lesion by indomethacin. Pretreatment with genistein significantly improved ulcer indexes and increased the level of NO, PGE_2_ and SOD activity and significantly decreased MDA, levels of TNF-α and MPO activity, and expression of MMP-9 was negatively regulated [126]. In addition, the association of genistein with *N*-acetylcysteine was evaluated, showing a significantly better gastroprotective effect than in individual administration [127].

#### 2.5.2. Biochanin A

Biochanin A (5,7-dihydroxy-3-(4-methoxyphenyl)chromen-4-one) (Figure 6) is an O-methylated isoflavone found mainly in soybeans and red clover and has several effects, including anti-inflammatory, anti-cancer and neuroprotective [128]. It showed gastroprotective activity (25 and 50 mg/kg, p.o., rat) in the ethanol model, which was attributed to increased NO level and SOD activity and decreased MDA, with omeprazole (20 mg/kg) as a reference group [129].

### 2.6. Furanoflavonoids

Furanoflavonoids are flavonoids possessing a furan group. An example of such a compound is karanjin [130].

#### Karanjin

**Figure 7:**
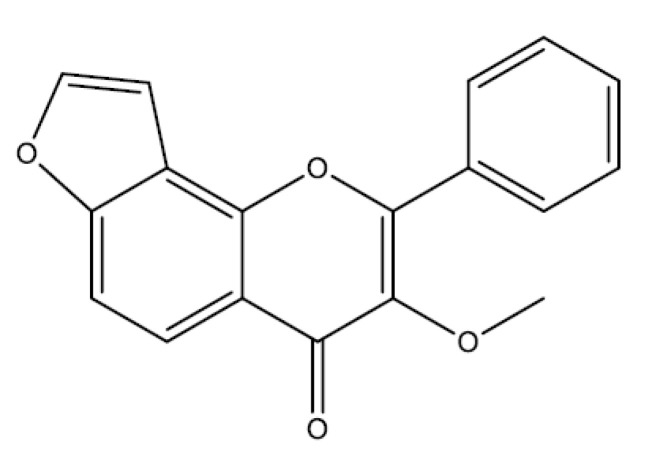
Furanoflavonoids (karanjin) with gastroprotective and/or anti-ulcer activity.

Karanjin (3-methoxy-2-phenylfuro [2,3-h] chromen-4-one) (Figure 7) is a furanoflavonoid isolated from *Pongamia pinnata* and showed neuroprotective [131] and antitumor effects [132] in experimental models. Karanjin (10 and 20 mg/kg) and omeprazole (20 mg/kg) showed gastroprotective activity in the ethanol and stress model, inhibited oxidative stress, with reduced levels of lipid peroxidation and increased activity of antioxidant enzymes (CAT, GPx, and SOD). In addition, karanjin negatively modulated the activity of H^+^-K^+^-ATPase in vitro (0.0273–0.192 mMol/L) and in vivo (in rats) [133].

### 2.7. Biflavonoids

Molecules formed by dimers of flavonoids are classified as biflavonoids [134]. Most naturally occurring biflavonoids are dimers of flavones and flavanones. These monomers can be of the same molecules or of different types of flavonoids [41].

#### Kolaviron

**Figure 8:**
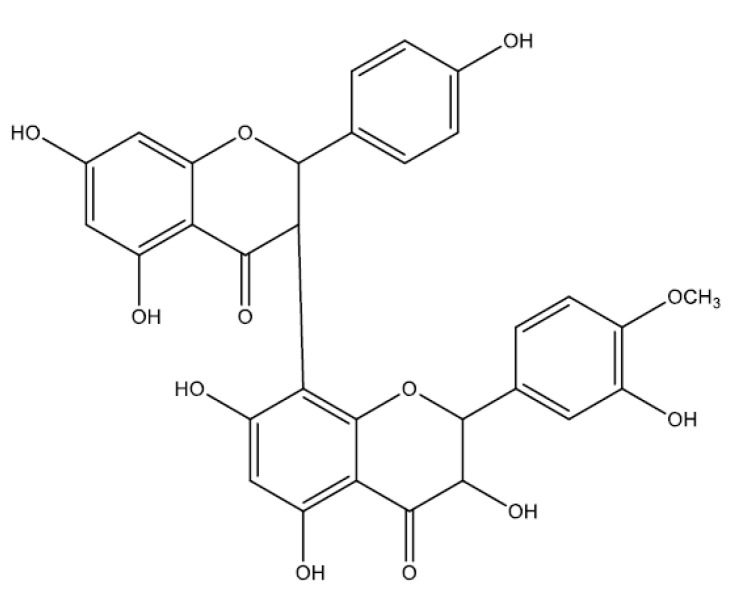
Biflavonoids (kolaviron) with gastroprotective and/or anti-ulcer activity.

Kolaviron (Figure 8), a biflavonoid (a complex of two compounds), has been identified as the active compound in the seed of *Garcinia kola*, known for its neuroprotective [135], cardioprotective [136], hepatoprotective and protective kidney activities [137]. Kolaviron (100 and 200 mg/kg, p.o., rat) showed a potential gastroprotective activity in different experimental models of peptic ulcer: immobilization and cold stress, NSAIDs, ethanol, pyloric ligation, and ischemia-reperfusion. This activity was related to antioxidant, cytoprotective, and antisecretory effects, with reduced levels of MDA, increased levels of NO, and gastric mucus. In addition, it had an inhibitory effect on H^+^-K^+^-ATPase activity in vitro with IC_50_ of 0.0744 mMol/L [138,139].

### 2.8. Other Flavonoids

#### Silymarin

**Figure 9:**
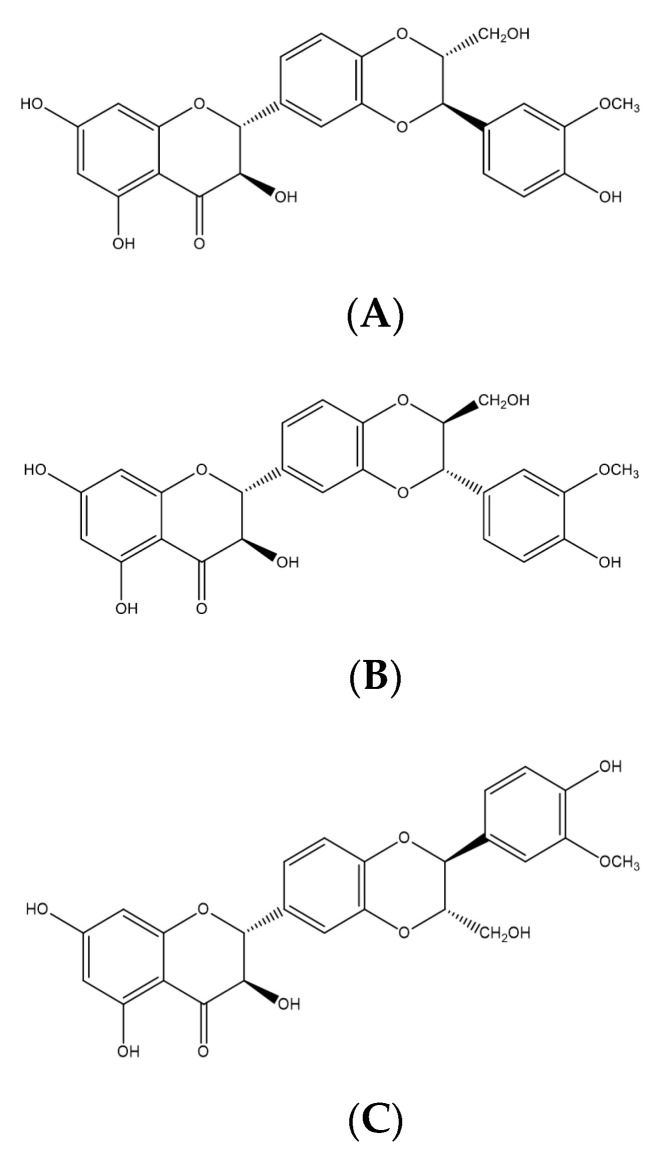

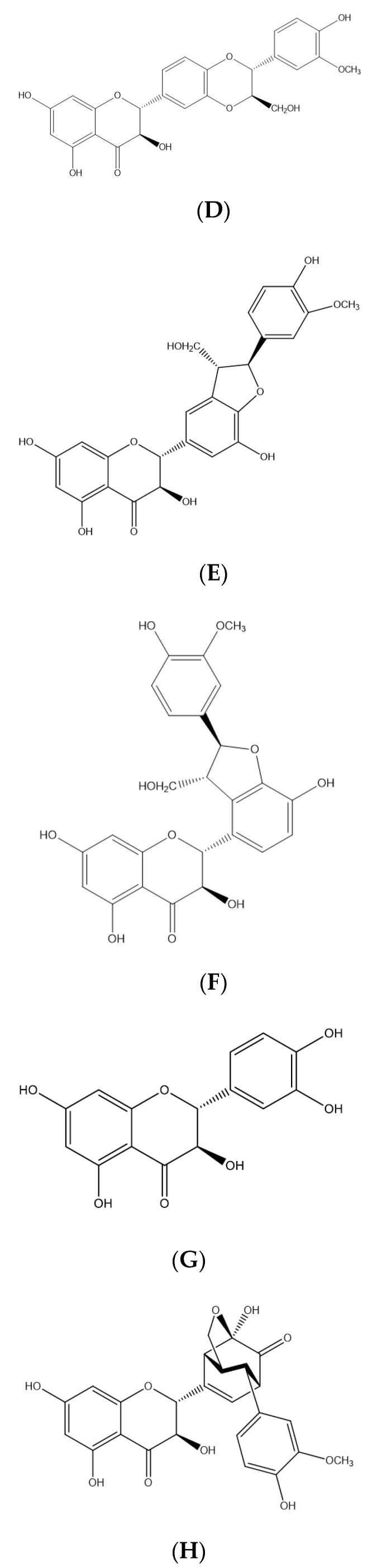
The structure of the components of Silymarin. Legend: (**a**) silibinin A (**b**) silibinin B, (**c**) isosilybin A (**d**) isosilybin B, (**e**) silychristin, (**f**) isosilychristin, (**g**) silydianin and (**h**) taxifolin.

Silymarin, a phytocomplex extracted from the medicinal plant *Silybum marianum* (“milk thistle”), is widely known for its hepatoprotective functions [140]. In addition, they have a protective effect against injuries to the brain [141], heart [142], and kidneys [143]. It is a biflavonoid, and consists of several flavonolignans (silibinin A and B, isosilybin A and B, silychristin, isosilychristin, and silydianin) and some flavonoid compounds, mainly taxifolin and quercetin [144]. The diastereoisomers silibinin A and B are the main actives ingredients of silymarin [145] (Figure 9). Investigations were carried out to determine the anti-ulcer effects of silymarin in different experimental models: cold restriction stress [146], ischemia-reperfusion [147], ligature of pylorus [148], absolute ethanol [149] and NSAIDs [150]. This gastroprotective effect (50 mg/kg, p.o., rat) is mediated by suppression of gastric inflammation with reduced MPO activity, levels of TNF-α, IL-6 and expression of NF-κB. It involves antioxidant and cytoprotective defense mechanisms by inhibiting lipid peroxidation and increasing the activity of the enzymes, GPx and SOD, as well of Nfr_2_ [149,150]. These results are summarized and expressed in Table 1.

## 3. Discussion

It was possible in this review to list a collection of substances with antiulcer activity. Flavonoids are among the antiulcer agents for which gastroprotective and anti-ulcerogenic efficacy have been largely confirmed in vivo and in vitro assay.

Ethanol is widely used as an ulcerogenic agent in the evaluation of gastroprotective effects in experimental models, mainly due to the fact that alcohol is one of the most common causes of gastric ulcers in humans [151,152], causing damage to the gastric mucosa such as necrotic lesions, hemorrhage, and ulcerations [153], leading to oxidative stress that may result in the overproduction of reactive oxygen species (ROS) and lipid peroxidation [154,155,156]. Alcohol absorption occurs throughout the gastrointestinal tract. After absorption, ethanol is metabolized to acetaldehyde by the action of the alcohol dehydrogenase enzyme and later converted to acetic acid, which has cytotoxicity to gastric cells [151].

In addition, the direct contact of ethanol in the gastric mucosa results in the infiltration of polymorphonuclear cells, which are important releasers of ROS, increasing the production of pro-oxidative substances and pro-inflammatory molecules [152,154]. Studies have shown that pro-inflammatory cytokines, such as TNF-α, IL-1β, and IL-6 play important roles in the formation of gastric ulcers [157]. Ethanol is also capable of decreasing the levels of NO, which is an important mediator of physiological processes of the gastric mucosa, such as the maintenance of blood flow, thus leading to the development of hemorrhagic lesions and consequently solubilization of gastric mucus [152,154]. Flavonoids such as kaempferol [46], diosmin [92], nobiletin [103], hesperidin [119], and baicalein [81] reduced ethanol-induced ulcerations in rats, significantly protecting the gastric mucosa against this inducing agent, inferring that these phytochemicals have a gastroprotective effect.

The stress-induced gastric ulcer model has been extensively studied over time. Stress is a consequence of physiological changes when the body or mind is exposed to pressure situations. The damage to the gastric mucosa induced by stress can be acute, erosive, and inflammatory; it is related mainly to changes in microcirculation and hypoxia. Mucosal lesions are usually superficial and asymptomatic, but they can extend to the submucosal and muscular layers, breaking larger vessels and causing evident and clinical significance [158,159,160]. The flavonoids rutin [65], hesperidin [120], and kolaviron [138,139] have gastroprotective effects in this model, possibly being related to an anti-secretory or cytoprotective effect.

Another important ulcer induction mechanism is through NSAIDs. They exert a local action in gastric mucosa by destabilizing the layer of membrane phospholipids causing reperfusion of the H^+^ ions and tissue damage [161,162]. Systemically, they exert their effects by inhibiting isoforms of COX-1 and COX-2 and are one of the most common causes of peptic ulcers, reducing the synthesis of cytoprotein prostaglandins and consequently decreasing the secretion of mucus, bicarbonate, and maintenance of adequate blood flow [163,164]. Indomethacin is one of the NSAIDs best known for experimentally inducing gastric ulcers [165].

Isoorientin [98], chrysin [89], morin [60], baicalin [83], and genistein [125] have been shown to protect the gastric mucosa from damage induced by NSAIDs, suggesting that flavonoids can act by a cytoprotective mechanism (for example by stimulating prostaglandins and mucus and bicarbonate secretion), increasing the protective factors and maintaining the integrity of the gastric mucosa. Thus, these flavonoids could improve the therapeutic effectiveness of NSAIDs by delaying the gastrointestinal side effects associated with these drugs.

The pylorus ligation test is an experimental model of acute induction of gastric ulcers that provides important information about the effect of substances on gastric secretion [166]. This procedure activates the vagal reflex, thus, the mechanoreceptors located in the pyloric region start to stimulate the secretion of gastric acid through the neuronal, endocrine, and paracrine pathways. Thus, the different secretagogues activate the proton pump, increasing acid secretion [167]. The distention of the gastric mucosa caused by obstruction of the pylorus increases the release of acetylcholine (Ach) by the vagus nerve, which will act directly on G cells and parietal cells, stimulating the secretion of gastrin and histamine, respectively [168].

Besides, the pyloric ligation model mimics chronic constipation due to stasis of gastric motility, causing the acidic content to remain in contact with the gastric mucosa for a long time. Exposure of the gastric lumen to this accumulation of acid secretion promotes self-digestion of the gastric mucosa and loss of integrity of the mucosal barrier, leading to ulcer instability [167]. In this review, the kolaviron [138,139] and baicalein molecules [81] were evaluated against this induction method, presenting a gastroprotective effect that can be explained by a possible antisecretory activity (decreasing the production of H^+^).

Another method of inducing ulcers is ischemia and reperfusion. During the ischemic process, there is an increase in reactive oxygen and nitrogen species and inflammatory mediators that are disseminated during the reperfusion period causing damage to the mucosa [169]. Kolaviron had its gastroprotective effect evaluated in this method [138,139], suggesting an antioxidant effect, increasing the activity of antioxidant enzymes (SOD, CAT, GPx), and/or GSH levels in gastric mucosa tissue; it can also increase the levels of NO, which acts to maintain blood flow in the mucosa.

In addition to the acute ulcer induction models, there are the chronic ulcer models, such as those induced by acetic acids. The lesions produced by acetic acid are like human ulcers in terms of location, severity, chronicity, and the healing process [170]. Their development involves changes in the levels of PGE_2_, NO, growth factors, and cytokines, as well as changes in the microcirculation and the mucus adhesion pattern [171]. Rutin [65], hesperdidin [121], chrysin [90], and baicalin [85] showed antiulcerogenic and healing activity in this model; it can be inferred that flavonoids were able to accelerate the healing of ulcers, thereby being an important mechanism to reduce disease recurrences.

Flavonoids protect the gastric mucosa in several experimental models of ulcers and their action is related to many mechanisms, such as cytoprotective, anti-secretory, immunoregulatory, antioxidants and *H. pylori* inhibitors.

The antioxidant mechanism is important in the ability to strengthen the defensive factors of the gastric mucosa. The antioxidant mechanism of action of flavonoids includes: (1) free radical scavenging; (2) chelating metal; (3) inhibition of enzymes associated with the generation of free radicals; (4) stimulation of endogenous antioxidant mechanisms [172,173]. Studies showed that flavonoids rutin [63], diosmin [92], chrysin [89], isoorientin [98], and naringenin [107] increase the activities of antioxidant enzymes CAT, SOD, and GPx, protecting the gastric mucosa against oxidative stress.

In addition, Nrf2 plays a significant role in protecting the gastrointestinal tract from oxidative damage [174]. Nrf2 induces the expression of antioxidant defense proteins, including hemoxigenase-1 (HO-1) [175] and negatively regulates NFκB, thereby inhibiting pro-inflammatory signaling [176]. Studies show that the increase in Nrf2 expression plays an important protective role against gastric ulcers induced by ethanol and other harmful agents [174]. Flavonoids such as catechin [73], quercetin [55], and silymarin [149,150] increase Nrf2 expression.

Several studies show a positive correlation between the severity of gastric ulcer and the increase in pro-inflammatory cytokines (IL-1β, IL-6, and TNF-α), associated with a reduction in anti-inflammatory cytokines (IL-10), influencing inflammatory scores [177]. Many flavonoids regulate cytokines, such as nobiletin [103], naringin [107], chrysin [89] and kaempferol [46]. Other factors are also important points of evaluation of the inflammatory process, such as the expression of adhesion molecules, ICAM-1 and P-selectin, and inducible endothelial cell surface molecules involved in leukocyte adhesion [178]. Quercetin [55] and morin [60] modulate these molecules. Thus, these flavonoids can reduce the inflammatory process associated with ulcerative lesions, reducing the severity of the disease.

The damage of the gastric mucosa is mainly associated with pepsin activity and hypersecretion of HCl by the action of H^+^-K^+^-ATPase [179,180]. Flavonoids such as karanjin [133], rutin [66], kolaviron [139], baicalein [81], quercitrin and afzelin [68] inhibited the H^+^-K^+^-ATPase in experimental models in vitro and in vivo (in rodents), presenting anti-secretory effect.

Other flavonoids, such as epicatechin [72], epigallocatechin gallate [70,71], kaempferide [48], kolaviron [138], hesperidin [120,121], baicalein [81], and chrysin [89], increase gastric mucus. The mucus is secreted in a regulated manner by epithelial cells on the gastroduodenal surface and consists mainly of water and a mixture of phospholipids, glycoproteins (mucin), and HCO_3_^−^. The mucus maintains a pH gradient between 6 and 7 along the surface of epithelial cells in relation to pH 1 to 2 on the gastric luminal surface, preventing the proteolytic action of pepsin and HCl [4]. Mucus is formed by mucin granules which, when released by exocytosis, react with water and form viscous fluid, forming a physical barrier to external aggressors [181]. Studies show that even if low acid secretion occurs, weakening the mucus barrier can cause damage to the stomach wall [5]. Thus, these flavonoids act to increase the mucosal defense factors, protecting epithelial cells against the action of enzymes digestive, acid, abrasion of food particles and other aggressors.

NO is important in maintaining the integrity of the gastric mucosa since it is involved in cytoprotection, stimulating mucus and bicarbonate secretion, increasing the blood flow, and angiogenesis [182]. NO is synthesized by three different forms of nitric oxide synthase (NOS), which are iNOS, neuronal (nNOS) and endothelial (eNOS) synthase. nNOS and eNOS synthase contribute to the normal function of the gastrointestinal tract, since inhibition of these enzymes results in disorders of motility, blood flow and acid secretion. Under certain pathological conditions, iNOS produces relatively large amounts of NO contributing to mucosal damage and inflammatory process [183]. Flavonoids epigallocatechin gallate [70,71], rutin [64], morin [60] and genistein [125] modulate iNOS activity and cause overexpression of this enzyme isoform, protecting the mucosa from pro-oxidants damage.

NO’s actions can be mediated by soluble guanylyl cyclase (GC), resulting in an increase in cGMP that can modify the properties of ion channels such as K_ATP_. NO-cGMP-K_ATP_ axis has gastroprotective effects, and since the efflux of potassium promotes the blocking of voltage-sensitive calcium channels, it leads to the relaxation of smooth muscles, favoring blood flow and the arrival of growth factors and cell renewal that facilitate the ulcer healing process [184]. The gastroprotective effect of baicalein involves the NO-cGMP-K_ATP_ pathway, accelerating the healing of ulcers [81].

Prostaglandins are lipid mediators synthesized by the enzyme, COX. There are two main isoforms of COXs, with COX-1 (constitutive) responsible for maintaining baseline levels of PGs and COX-2 (inducible) involved in inflammatory processes. PGs play a central role in the defense of the gastric epithelium, by inhibiting acid secretion, stimulating mucus and HCO_3_^−^ secretion and increasing blood flow [185]. Many flavonoids exert gastroprotective effects through mechanisms that involve prostaglandins and the enzymatic activity of the COX, such as pinostrobin [109], chysin [90], and hesperidin [119], strengthening the defense mechanisms of the mucosa or slowing down the inflammatory process.

*H. pylori* causes inflammatory gastritis and has been considered a major contributor to the etiology of peptic ulcer disease, due to its virulence factors such as flagellin, urease, CagA and VacA. VacA is an exotoxin that induces in the epithelial cell the formation of intracytoplasmic vacuoles and the destruction of mitochondria with cytochrome C (Cit C) release and cell death by apoptosis. Cag A is highly immunogenic and induces a strong inflammatory response. Urease is associated with adaptation and flagellin with adhesion of microorganism [186].

Quercetin [56], kaempferol [47], and baicalein [82], decrease *H. pylori*-related inflammation, reducing TNF-α, IL-1β, and IL-8. In addition, kaempeferol [47], baicalein [82], and baicalin [82,84], act on different virulence factors, reducing the expression of the CagA and VacA genes. Baicalein [82] also promotes a decrease in the capacity of adhesion and invasion of this microorganism and baicalin [82,84], exerts an inhibitory effect on multidrug-resistant strains, significantly reducing the MICs of the antimicrobials amoxicillin and tetracycline when used in combination. This effect was related to the reduced expression of the hefA gene, which is related to efflux pumps, an important mechanism of drug resistance. Thus, this flavonoid can assist in the treatment of resistant *H. pylori* in a complementary way to existing antimicrobials.

Some studies have assessed apoptosis markers in models of gastric ulcer as caspase-3, Cit C, and Bcl-2. The apoptosis cascade is another important pathway involved in the pathogenesis of gastric ulcers and is also correlated with oxidative stress and the inflammatory response. Caspase-3 is the main executing enzyme, activated by the mitochondrial pathway and the death receptor. In the mitochondrial pathway, with ROS and TNF-α breaking the balance between pro and anti-apoptosis factors, Cit C is released from mitochondria into the cytosol, followed by caspase-9 and activation of caspase-3 [187]. Flavonoids diosmin [92], morin [60], chrysin [90] and naringin [107] modulate these markers apoptotic, protecting gastric tissue from inducing cell death.

When the cells of the gastric epithelium are subjected to some stress conditions, thermal shock factor 1 (HSF1) is transcribed, which in turn induces the production of heat shock proteins (HSPs), especially the HSP-70 chaperone, which prevents cells from entering apoptosis by preventing intracellular protein denaturation. Disturbance in the metabolism of HSP can also induce mucosal damage in response to ROS [188]. Thus, some studies have been dedicated to the evaluation of these proteins. Flavonoid catechin modulates HSP-70 in models of the gastric ulcer [72], reaffirming that some flavonoids can prevent cells from entering apoptosis, protecting them from the mechanisms of cell death.

One of the factors involved in the structuring of the gastric mucosa is matrix metalloproteinases (MMP). MMPs are a family of enzymes that are responsible for the degradation of the extracellular matrix and tissue remodeling. MMPs are important in the pathogenesis of ulcers, acting at the beginning of the formation of injuries, as well as in the healing process. MMP-9 is expressed by a few cells in a normal situation, but this isoform is highly inducible in inflammatory situations. Thus, MMP-9 is an important marker in the process of inflammation and ulcerations in the gastrointestinal tract [189]. Chrysin [90] and quercetin [53] promote a reduced expression of MMP-9 in ulcer models, which is considered a positive factor, as this enzyme causes damage by acting directly on tissue.

Angiogenesis is an essential element for the healing of gastric ulcers, as it contributes to the reepithelization and mucosal restitution through a reconstruction of the microvascular network, favoring the supply of blood flow, oxygen, and nutrients. VEGF plays an important role in the healing of gastric ulcers, stimulating the neovascularization [178]. Thus, the increased expression of VEGF and FGF are important markers in the healing process. Studies show that chrysin flavonoid has this effect [90], presenting a pro-angiogenic effect and favoring the reepitalization process, thus, it plays a healing role in the injured gastric epithelial cells.

Over the past 10 years, research on the anti-ulcerogenic mechanism of action of flavonoids has involved the multifaceted study of the disease, including damage to the gastric mucosa directly associated with extracellular matrix (MEC) degradation, in which matrix metalloproteinases (MMPs) play a crucial role. In the past 10 years, several animal studies with gastric ulcers have focused on the role of MMP-2 and MMP-9.

Another facet that emerged in these past 10 years of studies involved the study of Nrf2, a transcription factor directed to its ability to induce the expression of antioxidant defense, which protects the gastrointestinal tract against oxidative damage. In recent times, the apoptosis cascade has become an important pathological pathway to study gastric ulcers. This signaling cascade is correlated with oxidative stress and the inflammatory response and, in the last 10 years, some studies have demonstrated the contribution of flavonoids to caspase-mediated anti-apoptosis and angiogenesis.

## 4. Materials and Methods

The antiulcer activity of the flavonoids was researched using reference databases such as PubMed and Science Direct and keywords: gastroprotective or antiulcer or antiulcerogenic activity and flavonoids. Studies published from January 2010 to January 2020 were selected from the reference databases. References were consulted for details of the experimental models used to test the activity of the substances, dose, route of administration, tested organism, inducing agent, and effect. A survey was also carried out in the literature on the peptic ulcer and the general aspects of flavonoids. We chose to only include studies conducted with isolated flavonoids and exclude those in which flavonoids were components of crude extract or fractions to avoid the possible effects of interactions (synergisms and/or antagonisms between compounds).

## 5. Conclusions

In summary, it was possible to observe that the flavonoids were able to protect the gastric and/or duodenal mucosa against different induction models that mimic the ulcer in men (ethanol, NSAIDs, stress and pyloric ligation), through multiple mechanisms of action such as cytoprotectors (increased mucus), antioxidants (increased activity of SOD and CAT enzymes and GSH levels), immunoregulatory (reduction in proinflammatory cytokines and increase in anti-inflammatory cytokines), antisecretory (reduction in H^+^) and anti-*H. pylori*. Thus, they can potentially be used as preventive and complementary drugs or as dietary supplements to prevent the development of peptic ulcer and its episodes of recurrence and/or assist in the traditional treatment of ulcerative lesions. Many products of natural origin, especially composed of plant foods and plants, often referred to as complementary and alternative medicines, such as nutraceuticals and herbal medicines, respectively, have stood out for their therapeutic properties, which can assist in the management of many diseases.

## Figures and Tables

**Table 1:** Flavonoids with gastroprotective and/or anti-ulcer activity.

Compound	Natural and/or Food Source	Experimental Protocol/Dose	Route Administration/Organism Test	Effect or Mechanism	Reference
**Flavonols**					
**Quercetin**	Food source: onions, broccoli, apple, cherry and grape	in vivo Ethanol—2.5 mg/kg	(p.o.)/Rat	↓ MMP-9, iNOS, MPO ↑GSH	[53]
		in vivo NSAIDs (indomethacin)—50 and 100 mg/kg	(p.o.)/Rat	↑ Nrf2, SOD, GPx ↓ ICAM-1, MPO, P-selectin	[55]
		in vitro NSAIDs (indomethacin)—0.0331 mMol/L	Caco-2 cell	↓ NF-κB, IL-8	[55]
		in vitro anti-*H*. *pylori*—0.000212–0.423 mMol/L	Cell culture	Inhibition	[56]
		in vivo anti-*H. pylori*—25 mg/kg	(p.o.)/Mice	↓ TNF-α, IL-1β	[56]
**Kaempferol**	Food source: broccoli, cabbage, beans, leeks, tomatoes, strawberries, grapes and propolis	in vivo Ethanol—40, 80 and 160 mg/kg	(p.o.)/Mice	↓ MPO, TNF-α, IL-1β, IL-6 ↑ NO	[46]
		in vitro anti-*H. pylori*—0.0015625 to 0.2 mMol/L	AGS cells	↓ CagA, VacA, TNF-α, IL-1β, IL-8	[47]
**Kaempferide**	Propolis	in vivo Pylorus ligature—3 mg/kg	(i.g)/Mice	↓ pH, H^+^ concentration, secretion of volume, pepsin activity	[48]
		in vivo HCl/Ethanol—3 mg/kg	(i.p)/Mice	↑ SOD, CAT, GST, mucus ↓ MPO	[48]
**Morin**	Species of *Moraceae* family	in vivo NSAIDs (indomethacin)—50 mg/kg	(p.o.)/Rat	↓ MPO, NF-κB, TNF-α, iNOS, ICAM-1, IL-6, caspase-3 ↑ PGE_2_, SOD	[60]
**Quercitrin**	*Solidago chilensis*	in vivo HCl/Ethanol—1, 38 mg/kg	(p.o.)/Mice	↑ GSH ↓ MPO	[68]
		in vitro Proton pump activity—0.00223 to 0.223 mMol/L	Cell culture	Inhibition	[68]
**Afzelin**	*Solidago chilensis*	in vivo HCl/Ethanol—0.026 and 0.078 mg/kg	(p.o.)/Mice	↓ MPO	[68]
		in vitro Proton pump activity—0.00231 to 0.231 mMol/L	Cell culture	Inhibition	[68]
**Rutin**	Food source: tomatoes, orange, carrots, sweet potatoes, black tea, and apple peels	in vivo NSAIDs (indomethacin)—200 mg/kg	(p.o)/Rat	↑ GSH, SOD ↓ MPO	[63]
		in vivo-Ischemia and reperfusion—50, 100, and 200 mg/kg	(p.o)/Rat	↓ iNOS, MPO, MDA	[64]
		in vivo Ethanol—20, 40 and 80 mg/kg	(p.o)/Rat	↓ MDA ↑ GPx	[65]
		in vivo Acetic Acid—20, 40 and 80 mg/kg	(p.o)/Rat	↓ MDA ↑ GPx	[65]
		in vitro Proton pump activity—IC_50_ of 0.0590 mMol/L	Cell culture	Inhibition	[66]
**Flavanols**					
**Catechin**	Green tea	in vivo NSAIDs (ketoprofen)—25 mg/kg	(p.o)/Rat	↑ GPx, GR, Nrf2	[73]
**Epicatechin**	Green tea	in vivo Pylorus ligature—25 and 50 mg/kg	(p.o)/Rat	↓ H^+^ secretion	[72]
		in vivo Ethanol—25 and 50 mg/kg	(p.o)/Rat	↑ Mucus, SHs, NO, SOD, HSP-7	[72]
**Epigallocatechin gallate**	Green tea	in vivo NSAIDs (indomethacin)—0,5, 1, 2, 3 and 5 mg/kg	(p.o)/Rat	↓ iNOS, MPO ↑ Mucin, PGE_2_	[70,71]
**Flavones**					
**Isoorientin**	*Gentiana triflora* and *Eremurus spectabilis*	in vivo NSAIDs (indomethacin)—25, 50, 100 mg/kg	(p.o)/Rat	↓ MDA ↑ GSH, SOD	[98]
**Chrysin**	*Passiflora incarnate, Oroxylum indicum, Matricaria chamomilla,* propolis	in vivo NSAIDs (indomethacin)—50 and 100 mg/kg	(p.o)/Rat	↑ Mucus, GSH, CAT, VEGF ↓ MDA	[89]
		in vivo Acetic Acid—10 mg/kg	(p.o)/Mice	↓ COX-2, MMP-9, caspase-3 ↑ EGF, COX-1	[90]
**Nobiletin**	Citrus fruits	in vivo Ethanol—5, 10 or 20 mg/kg	(p.o)/Mice	↑ PGE_2_, SOD ↓ TNF-α, IL-6	[103]
**Baicalein**	*Scutellaria baicalensis*	in vivo HCl/Ethanol—10, 30 and 100 mg/kg	(p.o)/Mice	↑ SHs, NO, GSH, mucus ↓ MPO	[81]
		in vitro Proton pump activity—0.0370 and 0.111 mMol/L	Culture cell	Inhibition	[81]
		in vitro anti-*H. pylori*—IC_50_ 0.331 mMol/L	AGS cells	↓ IL-8, Vac A, capacity of adhesion	[82]
**Baicalin**	*Scutellaria baicalensis*	in vivo Pylorus ligature—5, 10 and 15 mg/kg	(p.o)/Rat	↓ H^+^ secretion	[83]
		in vivo Acetic Acid—6.5 and 13 mg/kg	(p.o)/Rat	↑ SOD, GSH, GPx ↓ MDA, IL-8, TNF-α	[85]
		in vitro anti-*H. pylori*—IC_50_ 0.431 mMol/L	GES-1 cells	↓ IL-8, IL-1β, Vac A, urease, adhesion, hefA	[82,84]
**Diosmin**	Citrus fruits (lemon)	in vivo Ethanol—100 mg/kg	(p.o)/Mice	↓ TNF-α, NF-kB, MPO, caspase-3, Cit C, Bcl2 ↑ IL-10, GSH, GPx, PGE_2_, NO	[92]
		in vitro anti-*H. pylori*—CIM 0.822 mMol/L	Culture cell	Inhibition	[93]
**Flavanones**					
**Naringin**	Citrus fruits (orange, lemon)	in vivo Ethanol—100 and 200 mg/kg	(p.o)/Rat	↓ MDA, IL-6, TNF-α, caspase-3 ↑ GSH, SOD	[107]
**Pinostrobin**	*Boesenbergia rotunda*	in vivo Ethanol—20 and 40 mg/kg	(p.o)/Rat	↓ COX-2	[109]
**Flavanones**					
**Hesperidin**	Citrus fruits (orange, lemon)	in vivo NSAIDs (indomethacin)—150, 300 and 450 mg/kg	(p.o)/Rat	↑ GSH, SOD, CAT	[117]
		in vivo Ethanol—50 mg/kg	(p.o)/Rat	↓ COX-2, TNF-α, MDA ↑ SOD, CAT, GPx	[119]
		in vivo Stress—100 mg/kg	(p.o)/Rat	↑ GSH, SOD, CAT, mucin ↓ MDA	[120]
		in vivo Acetic Acid—3 and 10 mg/kg	(p.o)/Rat	↑ Mucin, GSH, SOD, CAT ↓ MPO	[121]
		in vitro anti-*H. pylori*—0.05–0.5 mMol/L	Culture cell	Inhibition	[122]
**Neohesperidine**	Citrus fruits (orange, lemon)	in vivo NSAIDs (indomethacin)—100 mg/kg	(p.o)/Rat	↓ COX-2, TNF-α, MDA ↑ GSH	[116]
**Isoflavonoids**					
**Genistein**	Soy-based foods (*Glycine max* or *Soy hispida*)	in vivo NSAIDs (indomethacin)—10 mg/kg	(p.o)/Rat	↓ TNF-α, MDA, iNOS ↑ SOD, PGE_2_	[125]
		in vivo NSAIDs (indomethacin)—10 mg/kg for 7 days	(p.o)/Rat	↑ NO, PGE_2_, SOD ↓ MDA, TNF-α, MPO	[126]
**Biochanin A**	Soy-based foods	in vivo Ethanol—25 and 50 mg/kg	(p.o)/Rat	↑ NO, SOD ↓ MDA	[129]
**Furanoflavonoids**					
**Karanjin**	*Pongamia pinnata*	in vivo Ethanol—10 and 20 mg/kg	(p.o)/Rat	↑ CAT, GPx, SOD ↓ MDA	[133]
		in vitro Proton pump activity-0.0273–0.192 mMol/L	Culture cell	Inhibition	[133]
**Biflavonoids**					
**Kolaviron**	*Garcinia kola*	in vivo Ethanol—100 and 200 mg/kg	(p.o)/Rat	↑ NO, mucus ↓ MDA	[138]
		in vitro Proton pump activity—IC_50_ of 0.0744 mMol/L	Culture cell	Inhibition	[139]
**Other flavonoids**					
**Silymarin**	*Silybum marianum*	in vivo Ethanol—50 mg/kg	(p.o)/Rat	↓ MPO, TNF-α, IL-6, NF-kB ↑ GPx, SOD, Nfr2	[149,150]

Legend: Bcl2-B-cell lymphoma-2; CagA-Cytotoxin associated gene A; CAT–Catalase; COX–Cyclooxygenase; GPx - Glutathione peroxidase; GSH-Glutathione; GST-Glutathione transferase; HSP-7-Heat shock protein-7; IL-10-Interleukin 10; IL-1β-interleukin 1 β; IL-6–Interleukin 6; IL-8–Interleukin 8; iNOS-Inducible nitric oxide synthase; MDA–Malondialdehyde; MMP-9-Matrix metalloproteinase; MPO-Myeloperoxidase; NF-κB-Nuclear factor-kappa B; NO–nitric oxide; Nrf2-Nuclear factor related to erythroid 2; NSAIDs-Non-steroidal anti-inflammatory; PGE_2_-Prostaglandin E_2_, SHs-Sulfhydryl compounds; SOD-Superoxide dismutase; TNF-α-Tumor necroses factor-α; VacA-Vacuolating cytotoxin; VEGF-Vascular endothelial growth fator.

## Abbreviations

Ach	Acetylcholine
Bcl-2	B-cell lymphoma-2
Cag A	Cytotoxin associated gene A
CAT	Catalase
COX	Cyclooxygenase
Cit C	Cytochrome C
eNOS	Endothelial nitric oxide synthase
FGF	Fibroblast growth factor
GCs	Soluble guanylyl cyclase
GPx	Glutathione peroxidase
GR	Glutathione reductase
GSH	Glutathione
GST	Glutathione transferase
HCl	Hydrochloric acid
HSP	Heat shock protein
HCO_3_^−^	Bicarbonate
HO-1	Heme oxygenase-1
H_2_O_2_	Hydrogen peroxide
HSF-1	Thermal shock factor 1
IL	Interleukin
iNOS	Inducible nitric oxide synthase
MDA	Malondialdehyde
MIC	Minimum inhibitory concentration
MMP	Matrix metalloproteinase
MPO	Myeloperoxidase
NF-kB	Nuclear factor-kappa B
NO	Nitric oxide
NOS	Nitric oxide synthase
Nrf2	Nuclear factor related to erythroid 2
NSAIDs	Non-steroidal anti-inflammatory
nNOS	Neuronal nitric oxide synthase
PGs	Prostaglandin
PGE_2_	Prostaglandin E_2_
PPIs	Proton pump inhibitors
ROS	Reactive oxygen species
SH	Sulfhydryl compounds
SOD	Superoxide dismutase
TAC	Total antioxidant capacity
TNF-α	Tumor necroses factor-α
VEGF	Vascular endothelial growth factor
VacA	Vacuolating cytotoxin
